# Linear Growth Trajectories in Early Childhood and Adult Cognitive and Socioemotional Functioning in a Guatemalan Cohort

**DOI:** 10.1093/jn/nxaa337

**Published:** 2020-11-26

**Authors:** María J Ramírez-Luzuriaga, John Hoddinott, Reynaldo Martorell, Shivani A Patel, Manuel Ramírez-Zea, Rachel Waford, Aryeh D Stein

**Affiliations:** Nutrition and Health Science Program, Laney Graduate School, Emory University, Atlanta, GA, USA; Division of Nutritional Sciences and Charles H. Dyson School of Applied Economics and Management, Cornell University, Ithaca, NY, USA; Nutrition and Health Science Program, Laney Graduate School, Emory University, Atlanta, GA, USA; Hubert Department of Global Health, Rollins School of Public Health, Emory University, Atlanta, GA, USA; Nutrition and Health Science Program, Laney Graduate School, Emory University, Atlanta, GA, USA; Hubert Department of Global Health, Rollins School of Public Health, Emory University, Atlanta, GA, USA; INCAP Research Center for the Prevention of Chronic Diseases, Institute of Nutrition of Central America and Panama, Guatemala City, Guatemala; Nutrition and Health Science Program, Laney Graduate School, Emory University, Atlanta, GA, USA; Hubert Department of Global Health, Rollins School of Public Health, Emory University, Atlanta, GA, USA; Nutrition and Health Science Program, Laney Graduate School, Emory University, Atlanta, GA, USA; Hubert Department of Global Health, Rollins School of Public Health, Emory University, Atlanta, GA, USA

**Keywords:** latent class growth analysis, linear growth trajectories, executive function, nonverbal fluid intelligence, socioemotional functioning

## Abstract

**Background:**

Growth faltering in early childhood is associated with poor human capital attainment, but associations of linear growth in childhood with executive and socioemotional functioning in adulthood are understudied.

**Objectives:**

In a Guatemalan cohort, we identified distinct trajectories of linear growth in early childhood, assessed their predictors, and examined associations between growth trajectories and neurodevelopmental outcomes in adulthood. We also assessed the mediating role of schooling on the association of growth trajectories with adult cognitive outcomes.

**Methods:**

In 2017–2019, we prospectively followed 1499 Guatemalan adults who participated in a food supplementation trial in early childhood (1969–1977). We derived height-for-age sex-specific growth trajectories from birth to 84 mo using latent class growth analysis.

**Results:**

We identified 3 growth trajectories (low, intermediate, high) with parallel slopes and intercepts already differentiated at birth in both sexes. Children of taller mothers were more likely to belong to the high and intermediate trajectories [relative risk ratio (RRR): 1.21; 95% CI: 1.15, 1.26, and RRR: 1.11; 95% CI: 1.07, 1.15, per 1-cm increase in height, respectively] compared with the low trajectory. Children in the wealthiest compared with the poorest socioeconomic tertile were more likely to belong to the high trajectory compared with the low trajectory (RRR: 2.24; 95% CI: 1.29, 3.88). In males, membership in the high compared with low trajectory was positively associated with nonverbal fluid intelligence, working memory, inhibitory control, and cognitive flexibility at ages 40–57 y. Sex-adjusted results showed that membership in the high compared with low trajectory was positively associated with meaning and purpose scores at ages 40–57 y. Associations of intermediate compared with low growth trajectories with study outcomes were also positive but of lesser magnitude. Schooling partially mediated the associations between high and intermediate growth trajectories and measures of cognitive ability in adulthood.

**Conclusions:**

Modifiable and nonmodifiable risk factors predicted growth throughout childhood. Membership in the high and intermediate growth trajectories was positively associated with adult cognitive and socioemotional functioning.

## Introduction

The period from conception to 2 y is crucial for neurobehavioral development (i.e., sensory–motor, cognitive–language, and social–emotional function), providing the foundation for lifelong health and well-being (1). In low- and middle-income countries, growth faltering, defined as lower length or height than expected for age and sex, also occurs during this period (2). Studies consistently show that growth faltering during the first 2 y of life is associated with poor cognitive development, reduced schooling, and adult economic productivity (3–6). However, associations of linear growth in early childhood with socioemotional outcomes in adulthood are understudied. Moreover, studies examining mediators of these associations have rarely been conducted.

Studies have used height-for-age *z* scores (HAZs) measured at 1 time point (or at most a limited number of time points) to examine associations of linear growth in childhood with human capital formation (4). Conditional growth variables, which assess deviations from a child's predicted rate of linear growth, have been utilized (5–7), but this method assumes that all children are drawn from a single underlying population for which a single set of parameters can be estimated, potentially masking heterogeneity in growth among subgroups of children.

In this study, we used prospectively recorded data over 50 y from a longitudinal cohort in Guatemala to identify groups of children with distinct trajectories of linear growth in early childhood and assess the influence of antenatal and postnatal exposures on growth trajectories. Next, we examined the association between growth trajectories and neurodevelopmental outcomes in adulthood. Finally, we assessed the mediating role of schooling on the association of growth trajectories with adult cognitive outcomes.

## Methods

### Study participants

Participants in this investigation were members of the Institute of Nutrition of Central America and Panama (INCAP) community-randomized food supplementation trial conducted between 1969 and 1977 (8, 9). Briefly, a total of 2392 children born between 1962 and 1977 were recruited in 4 villages in eastern Guatemala and were randomly assigned to receive a treatment drink (*atole*) or a control drink (*fresco*) twice a day for the duration of the study. *Atole* was a moderate-energy and protein drink supplement, whereas *fresco* was a low-energy drink with no protein. Participants have been followed up on several occasions. This article utilizes data collected in 2015–2017 and 2017–2019, at a mean cohort age of 44 and 47 y, respectively. Attrition through 2015–2017 has been published elsewhere (10). In 2017–2019, out of 1643 eligible participants presumed alive and living in Guatemala (68.7% of the original cohort), 375 declined to participate.

**FIGURE 1:**
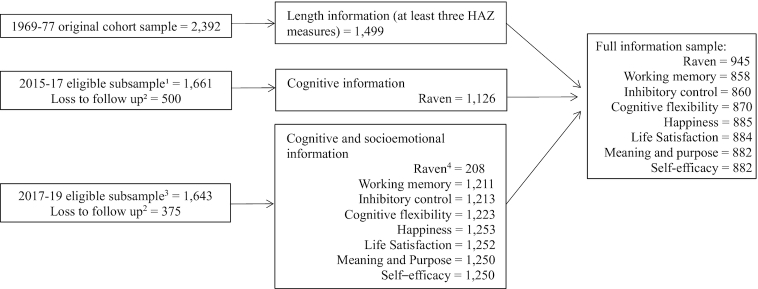
Participant flowchart. Inclusion criteria: participants who had ≥3 length measures in childhood and had ≥1 measure of cognitive ability or socioemotional functioning collected in either the 2015–2017 study wave or the 2017–2019 study wave. ^1^Out of 2392 participants, 369 died, 249 had migrated from Guatemala, and 113 were untraceable. ^2^Includes participants who either declined to participate or could not be contacted after several attempts. ^3^Out of 2392 participants, 385 died, 255 had migrated from Guatemala, and 109 were untraceable. ^4^Participants were administered a Raven's test in 2017–2019 if they had not completed it in the 2015–2017 wave. HAZ, height-for-age *z* score.

Our analytical sample is based on 1499 participants who had ≥3 length measures in childhood (0–84 mo) and had a measure of cognitive ability or socioemotional functioning collected in 2015–2017 or in 2017–2019 (**Figure 1**).

Participants provided written informed consent. The study was approved by INCAP and Emory University's Institutional Review Board at these 2 waves; the original study was conducted before the introduction of systematic ethical review of research.

### Measures

#### Growth trajectory measures

Length to the nearest 0.1 cm was measured at or near 15 d and at 3, 6, 9, 12, 15, 18, 21, 24, 30, 36, 42, 48, 60, 72, and 84 mo using length boards. To convert lengths to standing heights, we subtracted 1.0 cm from length measures obtained at 24 mo or younger (11). We converted heights into HAZs using the WHO's growth standards (12, 13).

#### Neurodevelopmental outcomes

In 2015–2017, nonverbal fluid intelligence was assessed using the Raven's Progressive Matrices test, modules A–C (14, 15). Previous applications of this test indicated that participants in this cohort did not progress past section C. In 2017–2019, participants were administered this test if they had not completed it in the 2015–2017 wave. Scores were computed as the sum of correct responses, with a maximum total score of 36. All other outcome measures were assessed during the 2017–2019 follow-up. We measured executive function using subtests of the NIH's Toolbox Cognition Battery. We used the List Sorting Working Memory Test to assess working memory capacity (16). Participants were asked to remember lists of illustrated pictures in size order from smallest to largest. Final scores were computed as the sum of correct responses across trials for a maximum score of 26. We assessed inhibitory control using the Flanker Inhibitory Control and Attention Test (17). The task requires respondents to focus on a central stimulus while inhibiting attention to the flanking stimuli surrounding it. We assessed cognitive flexibility using the Dimensional Change Card Sort Test (DCCS). Respondents are asked to switch between matching pictures by color and matching pictures by shape (17). For the Flanker and DCCS tests, we used the NIH Toolbox computed scores, which combine accuracy and reaction time using a 2-vector algorithm (18).

We assessed happiness using the Subjective Happiness scale, which consists of 4 items on a 5-point Likert scale. Final scores were computed as the mean of the 4 items (19). We used subscales of the NIH Toolbox Emotion Battery to assess life satisfaction, meaning and purpose, and self-efficacy (20). The scales consist of 5, 9, and 10 items, respectively, each on a 5-point Likert scale ranging from 1 (strongly disagree/never) to 5 (strongly agree/very often) (20). We computed final scores as the sum of items, after applying a 2-way imputation approach for missing items within each scale (21).

#### Early life characteristics

In 1969–1977, exposure to supplementation occurred at different age windows for each child, prenatally through maternal consumption and postnatally through breastfeeding and the child's own consumption. As described elsewhere (10), we categorized our study population into those exposed fully to supplementation from conception to age 2 y and those who received partial or no exposure during that period, based on the dates of availability of supplement and the child's date of birth, assuming a gestational age at birth of 266 d (38 wk). The interaction term between treatment assignment (*atole* or *fresco*) and categorization of age at exposure represents the differential effect of full exposure to *atole* from conception to age 2 y (22, 23). Information on maternal height, age at childbirth, and schooling was extracted from files created during the original study. A cumulative childhood household socioeconomic score was created using principal component analysis using information derived from village censuses conducted in 1967 and 1975 (24). In 2017–2019, school attainment of participants was ascertained by interview.

### Statistical analysis

#### Trajectories of attained HAZs

We used latent class growth analysis (LCGA) to identify distinct patterns of attained HAZs from birth to age 84 mo. LCGA is a special case of growth mixture modeling in which the variance and covariance of factors within each group are fixed to zero (25). To increase model stability, we restricted the identification of trajectories to participants with ≥3 HAZ measures collected between 0 and 84 mo. The optimal number of trajectory groups was determined by model fit statistics. The solution that best fitted the data was the one with the lowest Bayesian information criterion value, highest entropy, *P* values < 0.05 for Lo–Medell–Rubin likelihood ratio test and bootstrap likelihood ratio test, and the highest posterior probability (>0.70 for each class). In addition to model fit indices, we also considered the percentage of participants per trajectory group and interpretability (26). Trajectories were examined visually and arranged from high to low.

To account for the variability in the number of data points, which was more frequent at the younger ages, we conducted a sensitivity analysis restricting trajectories to children with available HAZ measures at birth and 48 mo and ≥1 HAZ measure collected between birth and 48 mo. To identify periods of growth more tightly linked to adult neurodevelopmental outcomes, and to examine the influence of exposure to nutritional supplementation from conception to age 2 y on linear growth, we identified growth trajectories using data collected from birth to age 35.9 mo, and separately from ages 36 to 84 mo among children with ≥3 HAZ measures in each interval.

#### Predictors of HAZ trajectories

We used multinomial logistic regression models to examine predictors of HAZ trajectory membership. Examined predictors included household socioeconomic tertile and maternal characteristics (age at childbirth, schooling, and height) considered to be antecedent to the pregnancy. Predictors of trajectories for ages 36–84 mo additionally included exposure to *atole* from conception to 2 y. All models controlled for fixed effects of birth village, birth year, and pooled models controlled for sex. Because a high proportion of participants were siblings, CIs accounted for clustering at the household level. We used multiple imputation for missing covariates (maternal age at childbirth, schooling, and height). The proportion of missing information was <5% for maternal age at childbirth and maternal schooling and <10% for maternal height.

We present pooled results when the number of identified trajectories across sexes does not differ, and we present sex-stratified results otherwise. We tested for equality of parameter estimates across sexes using postestimation tests.

#### Associations of HAZ trajectories with adult outcomes

We used 1-way ANOVA to compare cognitive and socioemotional mean scores across HAZ trajectories and linear regression models to examine associations of HAZ trajectory membership with cognitive and socioemotional scores in adulthood.

All models controlled for fixed effects of birth village, birth year, exposure to nutritional supplementation from conception to 2 y, maternal characteristics (age at childbirth, schooling, and height), and household socioeconomic status. Pooled models adjusted for sex. We assessed potential sex differences on examined associations by testing the interaction term between sex and growth trajectories. We accounted for clustering of children within family and used multiple imputation for missing covariates (discussed previously).

To assess the consistency of results with those using other methods to characterize growth, we conducted a sensitivity analysis using conditional growth measures and HAZs at 24 mo as predictors of study outcomes, adjusting for the same set of covariates previously described.

#### Mediation analysis

We used path analysis to evaluate the mediating role of schooling on the association of HAZ trajectories with cognitive scores using maximum likelihood estimation. We chose schooling as the mediator variable because it is a key component of the development of cognitive capacities (27), and previous studies in this cohort show that it is positively associated with adult measures of intellectual functioning (28). Models adjusted for fixed effects of birth village, birth year, exposure to *atole* from conception to 2 y, maternal height, and sex, and they accounted for clustering of participants within family.

We inferred statistical significance at *P *< 0.05, and all testing was 2-tailed. We used Mplus 8 for the identification of trajectories and mediation analyses. All other statistical analyses were conducted using STATA version 16 (StataCorp).

## Results

**FIGURE 2:**
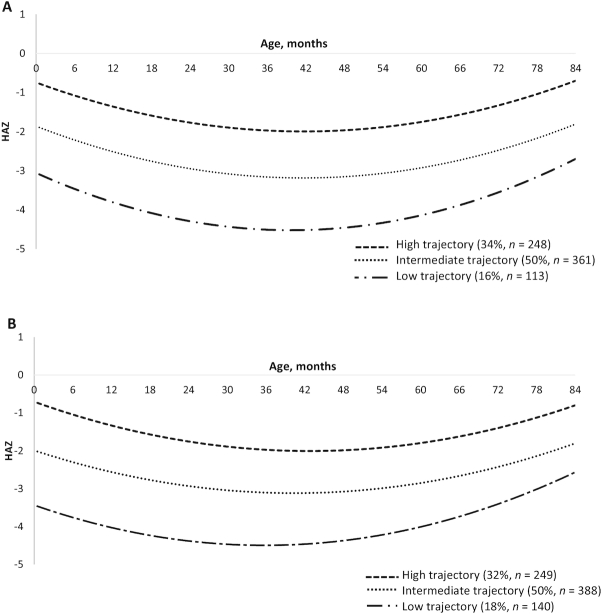
HAZ growth trajectories from birth to age 84 mo in females (A) and males (B). HAZ, height-for-age *z* score.

Descriptive statistics of the study population are presented in **Table 1**. We identified 3 HAZ trajectories in both sexes with parallel slopes and intercepts clearly differentiated at birth (**Supplemental Table 1**). Arranging trajectories as high, intermediate, and low, the estimated percentages were 34%, 50%, and 16% among females and 32%, 50%, and 18% among males, respectively (**Figure 2**). We also identified 3 trajectories in both sexes using data from the 0- to 35.9-mo period (**Supplemental Figure 1**, **Supplemental Table 2**) and 2 trajectories in females and 3 in males when using data from the 36- to 84-mo period (**Supplemental Figure 2**, **Supplemental Table 3**).

**TABLE 1:** Selected characteristics of study population by sex: INCAP longitudinal trial 1969–1977 (*n* = 1499)^1^

	Females (*n* = 722)	Males (*n* = 777)
Childhood household SES tertile, %		
Poorest	35.3	34.2
Middle	31.2	32.3
Wealthiest	33.5	33.5
Mother's age at childbirth, y	27.5 ± 7.2	27.4 ± 7.1
Mother's schooling, y	1.3 ± 1.6	1.3 ± 1.5
Mother's height, cm	148.5 ± 5.2	148.6 ± 5.2
Child's schooling, y	4.6 ± 3.5	5.4 ± 3.6
HAZ trajectory group, %		
Low	15.6	18.0
Intermediate	50.0	49.9
High	34.3	32.0

1 Values are means ± SDs or percentages. HAZ, height-for-age *z* score; INCAP, Institute of Nutrition of Central America and Panama; SES, socioeconomic status.

The distribution of children within each class remained similar whether HAZ trajectories were estimated using data at ages 0–84, 0–35.9, or 36–84 mo (**Supplemental Table 4**). When trajectories were derived restricting the sample to children with available HAZs at birth and 48 mo and ≥1 HAZ measure between birth and 48 mo, the derived trajectories remained similar to those derived using all data points (**Supplemental Figure 3**).

### Predictors of HAZ trajectories

Relative to the low trajectory, children with high or intermediate trajectories had taller mothers [relative risk ratio (RRR): 1.21; 95% CI: 1.15, 1.26, and RRR: 1.11; 95% CI: 1.07, 1.15 per 1 cm increase in height, respectively]. Children in the wealthiest compared with the poorest socioeconomic tertile were more likely to belong to the high or intermediate trajectory than the low trajectory (RRR: 2.24; 95% CI: 1.29, 3.88, and RRR: 1.45; 95% CI: 0.90, 2.31, respectively) (**Table 2**). Results using growth trajectories from the 0- to 35.9-mo period were similar (**Supplemental Table 5**).

**TABLE 2:** Predictors of HAZ trajectory membership at ages 0–84 mo (*n* = 1499)^1^

	Intermediate vs. low HAZ trajectory	High vs. low HAZ trajectory
Childhood socioeconomic tertile		
Middle vs. poorest	1.05 (0.72, 1.54)	1.24 (0.77, 1.99)
Wealthiest vs. poorest	1.45 (0.90, 2.31)	2.24 (1.29, 3.88)*
Mother's age at childbirth, y	0.98 (0.96, 1.01)	1.01 (0.98, 1.04)
Mother's schooling, y	0.97 (0.86, 1.10)	1.03 (0.89, 1.19)
Mother's height, cm	1.11 (1.07, 1.15)*	1.21 (1.15, 1.26)*

1 Values are relative risk ratios and 95% CIs. Low HAZ trajectory is the reference category. Estimates adjusted for sex and fixed effects of birth village and birth year. For missing covariates, multiple imputation techniques were used. CIs account for clustering at the mother level. HAZ, height-for-age *z* score. *P < 0.05.

Children exposed to *atole* from conception to age 2 y were more likely to belong to the high trajectory compared with the low trajectory in females during the 36- to 84-mo period (RRR: 2.99; 95% CI: 1.26, 7.11) (**Supplemental Table 6**). Tests of equality of parameter estimates across sexes did not reject the null hypothesis (*P *= 0.43).

### Associations of HAZ trajectories with adult outcomes

The distribution of participants with full trajectory data was similar between those with missing and those with available outcome measures (**Supplemental Tables 7–10**). Mean scores on cognitive tests obtained at ages 40–57 y were higher among participants in the high-HAZ trajectory at ages 0–84 mo (**Table 3**).

**TABLE 3:** Cognitive and socioemotional characteristics of the study population at ages 40–57 y, by childhood HAZ trajectory and sex^1^

	Women (*n* = 722)				Men (*n* = 777)			
	Low trajectory (*n* = 113; 16%)	Intermediate trajectory (*n* = 361; 50%)	High trajectory (*n* = 248; 34%)	*P* value^2^	Low trajectory (*n* = 140; 18%)	Intermediate trajectory (*n* = 388; 50%)	High trajectory (*n* = 249; 32%)	*P* value^2^
Cognitive measures								
Raven Progressive Matrices score (out of 36)	14.6 ± 4.5	15.2 ± 4.9	16.2 ± 5.0	<0.05	15.8 ± 4.6	18.1 ± 5.5	20.4 ± 6.7	<0.001
List Sorting Working Memory score (out of 23)	10.5 ± 3.8	11.2 ± 3.6	12.2 ± 3.6	<0.01	11.6 ± 3.4	12.8 ± 3.7	13.6 ± 4.2	<0.01
Flanker Inhibitory Control and Attentioncomputed score (out of 9)	5.3 ± 1.1	5.4 ± 1.1	5.5 ± 1.0	0.21	5.4 ± 1.0	5.8 ± 1.2	6.1 ± 1.2	<0.001
DCCS computed score (out of 9)	4.9 ± 1.9	5.0 ± 1.9	5.3 ± 1.9	0.25	4.6 ± 1.8	5.6 ± 1.9	6.0 ± 1.8	<0.001
Socioemotional measures								
Lyubomirsky Happiness score (out of 5)	4.0 ± 1.0	4.0 ± 1.0	4.2 ± 0.8	0.18	3.9 ± 0.8	4.2 ± 0.8	4.1 ± 0.8	0.05
Life Satisfaction score (out of 25)	18.2 ± 3.4	18.8 ± 3.3	18.7 ± 3.5	0.38	18.5 ± 3.7	19.2 ± 3.3	18.8 ± 3.5	0.23
Meaning and Purpose score (out of 45)	35.4 ± 3.6	36.3 ± 4.3	36.5 ± 3.8	0.13	36.4 ± 3.9	37.4 ± 3.8	37.8 ± 4.3	0.06
Self-efficacy score (out of 40)	30.8 ± 7.1	30.9 ± 7.2	31.2 ± 6.8	0.88	31.5 ± 7.1	32.2 ± 5.8	30.6 ± 6.5	0.08

1 Values are means ± SDs. DCCS, Dimensional Change Card Sort; HAZ, height-for-age *z* score.

2 Based on 1-way ANOVA.

We found evidence of heterogeneity by sex in pooled models examining associations of HAZ trajectories with cognitive outcomes, and thus we focus on sex-stratified results. We observed no significant interaction of sex and growth trajectories (*P *> 0.05) in models examining associations of HAZ trajectories with socioemotional outcomes.

Membership in the higher trajectory was associated with a 4.10-point increment in nonverbal fluid intelligence (95% CI: 2.49, 5.72), 1.73-point increment in working memory (95% CI: 0.50, 2.96), 0.65-point increment in inhibitory control (95% CI: 0.30, 0.98), and 1.21-point increment in cognitive flexibility (95% CI: 0.67, 1.75) in men. We observed no significant associations in women (**Table 4**). In models assessing socioemotional outcomes, membership in the higher trajectory was associated with a 1.09-point increment in meaning and purpose scores (95% CI: 0.22, 1.97) (**Table 5**). Associations of intermediate compared with low HAZ trajectories with cognitive and socioemotional outcomes were positive but of lesser magnitude.

**TABLE 4:** Associations of childhood HAZ trajectories with cognitive function at ages 40–57 y, by sex^1^

	Intermediate HAZ trajectory, β (95% CI)	High HAZ trajectory, β (95% CI)
Nonverbal fluid intelligence		
Women	0.09 (−1.07, 1.25)	0.48 (−0.84, 1.82)
Men	2.28 (1.02, 3.54)*	4.10 (2.49, 5.72)*
Pooled	1.19^2^ (0.33, 2.06)*	2.27^2^ (1.19, 3.34)*
Working memory		
Women	0.48 (−0.49, 1.46)	0.98 (−0.18, 2.16)
Men	1.18 (0.21, 2.15)*	1.73 (0.50, 2.96)*
Pooled	0.88 (0.21, 1.56)*	1.44 (0.58, 2.29)*
Inhibitory control		
Women	0.08 (−0.23, 0.39)	0.06 (−0.26, 0.39)
Men	0.49 (0.19, 0.77)*	0.65 (0.30, 0.98)*
Pooled	0.30 (0.08, 0.51)*	0.37^2^ (0.14, 0.61)*
Cognitive flexibility		
Women	−0.09 (−0.61, 0.42)	−0.01 (−0.59, 0.57)
Men	0.95 (0.47, 1.43)*	1.21 (0.67, 1.75)*
Pooled	0.47^2^ (0.12, 0.83)*	0.65^2^ (0.25, 1.06)*

1 Low HAZ trajectory is the reference category. For women and men, respectively, sample sizes were 523 and 422 for Raven's Progressive Matrices (nonverbal fluid intelligence), 468 and 390 for List Sorting Working Memory, 469 and 391 for Flanker Inhibitory Control and Attention, and 475 and 395 for Dimensional Change Card Sort (Cognitive flexibility). Models adjusted for fixed effects of birth village, birth year, exposure to supplementation from conception to age 2 y, the interaction term specifying exposure to *atole* from conception to age 2 y, maternal age at childbirth and maternal height (log transformed), maternal schooling, and household socioeconomic status in 1967–1975. Pooled models controlled for sex. For missing covariates, multiple imputation techniques were used. CIs account for clustering at the household level. HAZ, height-for-age *z* score. *P < 0.05.

2 *P *< 0.05 for the interaction of sex and HAZ growth trajectory.

**TABLE 5:** Associations of childhood HAZ trajectories with socioemotional functioning at ages 40–57 y^1^

	Intermediate HAZ trajectory, β (95% CI)	High HAZ trajectory, β (95% CI)
Happiness	0.14 (−0.03, 0.32)	0.20 (−0.00, 0.41)
Life satisfaction	0.69 (0.02, 1.37)*	0.54 (−0.25, 1.34)
Meaning and purpose	0.83 (0.07, 1.58)*	1.09 (0.22, 1.97)*
Self-efficacy	0.48 (−0.81, 1.78)	0.11 (−1.33, 1.55)

1 Low HAZ trajectory is the reference category. Sample sizes were 885 for Subjective Happiness scale, 884 for NIH Toolbox Life Satisfaction scale, 882 for NIH Toolbox Meaning and Purpose scale, and NIH Toolbox Self-Efficacy scale. Models adjusted for sex and fixed effects of birth village, birth year, exposure to supplementation from conception to age 2 y, the interaction term specifying exposure to *atole* from conception to age 2 y, maternal age at childbirth and maternal height (log transformed), maternal schooling, and household socioeconomic status in 1967–1975. For missing covariates, multiple imputation techniques were used. CIs account for clustering at the household level. HAZ, height-for-age *z* score. *P < 0.05.

Associations between HAZ trajectories over the 0- to 84-mo period and neurodevelopmental outcomes show similar effect sizes to associations of trajectories over the 0- to 35.9-mo period with the same outcomes. However, associations of trajectories over the 0- to 35.9-mo period with neurodevelopmental outcomes were considerably stronger than those between trajectories over the 36- to 84-mo period and the same adult outcomes (**Supplemental Tables 11** and **12**).

### Sensitivity analyses

Results using conditional growth variables showed positive associations of conditional lengths at birth and 24 mo with fluid intelligence. Furthermore, conditional length at birth, but not at later ages, was positively associated with inhibitory control and cognitive flexibility. Estimates were stronger for men than women (**Supplemental Table 13**). For socioemotional outcomes, birth length was positively associated with life satisfaction and meaning and purpose, whereas conditional length at 48 mo was positively associated with happiness and negatively associated with life satisfaction (**Supplemental Table 14**).

Results using HAZ at 24 mo as the predictor variable showed positive associations with fluid intelligence, working memory, inhibitory control, cognitive flexibility, and meaning and purpose, with estimates being stronger for men than for women (**Supplemental Table 15**).

### Mediation analyses

Mediation analysis showed that the indirect effect through schooling corresponded to 51% of the total effect of high HAZ trajectory (compared with low trajectory) on nonverbal fluid intelligence, 42% of the total effect of high HAZ trajectory (compared with low trajectory) on working memory, and 51% of the total effect of high HAZ trajectory (compared with low trajectory) on inhibitory control. Similar associations were observed between intermediate HAZ trajectory (compared with low trajectory) and nonverbal fluid intelligence, working memory and inhibitory control through schooling (**Table 6**). Models assessing mediation for cognitive flexibility did not converge.

**TABLE 6:** Mediation analyses of the association of childhood HAZ trajectories with cognitive scores at ages 40–57 y through schooling^1^

Type of effect	Nonverbal fluid intelligence	Working memory	Inhibitory control
Intermediate HAZ trajectory vs. low			
Total effect	0.21 (0.01, 0.42)*	0.22 (−0.01, 0.45)	0.29 (0.05, 0.53)*
Indirect effect via schooling	0.15 (0.06, 0.25)*	0.12 (0.05, 0.19)*	0.13 (0.05, 0.21)*
Direct effect	0.06 (−0.12, 0.24)	0.10 (−0.11, 0.32)	0.16 (−0.06, 0.38)
High HAZ trajectory vs. low			
Total effect	0.39 (0.13, 0.65)*	0.36 (0.06, 0.67)*	0.33 (0.06, 0.59)*
Indirect effect via schooling	0.20 (0.08, 0.33)*	0.15 (0.06, 0.25)*	0.17 (0.07, 0.27)*
Direct effect	0.19 (−0.05, 0.43)	0.21 (−0.08, 0.50)	0.16 (−0.09, 0.41)

1 Values are standardized coefficients (STDY in Mplus) and 95% CIs interpreted as the change in *y* in *y* SD units when *x* changes from 0 to 1. Sample size was 1073 for nonverbal fluid intelligence and 1049 for working memory and inhibitory control. Estimates adjusted for sex, maternal height, and fixed effects of birth village, birth year, and exposure to *atole* from conception to age 2 y. HAZ, height-for-age *z* score. *P < 0.05.

## Discussion

In this longitudinal cohort of Guatemalan adults, we documented that trajectories of attained linear growth (HAZ) from birth to age 7 y showed a gradient of positive associations with measures of intelligence and executive function at ages 40–57 y in men but not women. We also found that HAZ trajectories prior to age 7 y were not consistently associated with adult socioemotional outcomes. Furthermore, in agreement with previous literature, we found that associations of HAZ trajectories from birth to age 3 y with adult neurodevelopmental outcomes were considerably stronger than associations of HAZ trajectories at ages 3–7 y with the same adult outcomes. Our study further documents that schooling partially mediates the association of linear growth in childhood with cognitive function in adulthood. Because in this cohort men have completed more years of schooling than women, this difference could partially explain why associations between HAZ trajectories in childhood and cognitive outcomes in adulthood were observed only in men.

Adequate linear growth is an indicator of child health and a well-known determinant of childhood morbidity and mortality (29). Thus, it is possible that participants in the high and intermediate HAZ trajectories were healthier as children than those in the low HAZ trajectory. Healthy children are less likely to miss school and, as a result, may have more opportunities to receive stimulation from teachers and peers. Studies show that school environments may provide unique opportunities for children to improve and exercise their executive function skills, particularly for those coming from less supportive home environments (30, 31). Furthermore, it has been documented that children who complete more years of schooling score higher on measures of executive function (32, 33). Alternatively, it is also possible that children with higher cognitive abilities complete more years of schooling.

Our sensitivity analyses using conditional growth measures or HAZs at age 2 y as alternative representations of child growth were consistent with our findings using HAZ trajectories. Length at birth was positively associated with cognitive outcomes in adulthood among men, but associations were attenuated for conditional height at 2 and 4 y. Furthermore, HAZ at 2 y was positively and consistently associated with measures of cognitive ability in men. Associations of conditional growth measures, or HAZ at 2 y with socioemotional outcomes were less consistent.

Growth trajectories using LCGA and conditional growth variables offer advantages over single measures of growth. Both capture the influence of growth velocity at certain periods of development on outcomes of interest. Also, both methods allow to control for correlated measures of HAZ over time. However, conditional growth variables assume that the population is homogeneous with respect to how predictors associate with outcomes. In contrast, LCGA assumes that the population is heterogeneous with respect to how variables influence each other, allowing more nuanced results.

Our findings document that growth trajectories showed similar (parallel) slopes, which were primarily distinguished by the degree of linear growth faltering at birth (intercepts). The early differentiation of trajectories suggests that prenatal factors play a crucial role in establishing growth throughout childhood. Increased maternal height and higher household socioeconomic status predicted membership in the high growth trajectory across all age periods, whereas exposure to enhanced nutrition from conception to 2 y predicted higher growth trajectory membership over the 3- to 7-y period, highlighting the importance of early life nutrition for promoting child growth.

Our study findings suggest both modifiable and nonmodifiable risk factors for early life growth faltering. The influence of maternal height in offspring's linear growth is well established and is thought to reflect a complex interplay between genetic characteristics and persistent transgenerational effects of the environment in which the mother grew up and developed (34). Short women (<145 cm) are at higher risk of having small-for-gestational-age children, who in turn are more likely to suffer from stunted growth in early childhood (35, 36).

Regarding the positive associations between linear growth trajectories in childhood and neurodevelopmental outcomes in adulthood, our findings may be explained by increases in motor development during infancy. The acquisition of gross motor skills allows the child to access more stimulation through the exploration of the environment and interactions with caregivers, potentially influencing mental development (37). A study conducted in Bihar, India, among children aged 12–18 mo found indirect associations (through motor development) of length for age with mental development, including measures of executive function and personal–social development (38). The proposed mechanisms linking linear growth with delayed cognitive and socioemotional development suggest that growth faltering may reduce motor activity, limiting the child's ability to explore the environment and receive psychosocial stimulation (3), thus reducing opportunities for the development of language, socioemotional, and cognitive capacities (39). However, others have found that the attainment of gross motor skills is largely independent of variations in linear growth (40) and that the early acquisition of motor skills has no association with mental development (41). Other proposed mechanisms suggest reduced stimulation offered to short-stature children due to caregivers’ low expectations about their developmental potential (42). However, despite consistent associations between growth faltering and reduced cognitive function, recent academic debate has suggested that these associations are unlikely to be causally related (43, 44). A more plausible explanation is that linear growth may be a marker of early life neurobehavioral development (43), which remains intricately intertwined with both cognitive and socioemotional domains throughout the life course.

Our study has limitations. We did not control for early life variables, such as psychosocial stimulation and maternal mental health, that could potentially be associated with childhood linear growth and are likely to have an influence on cognitive and socioemotional functioning. Similarly, we did not include conditions in adolescence and adulthood that could potentially be related with childhood linear growth and likely to have a bearing on adult socioemotional functioning. Also, sensitivity analyses using conditional growth variables were restricted to participants for whom we had data on birth weight and on length at age 4 y, reducing the sample size substantially. This is likely to be the explanation for the unexpected negative association found between conditional length at 48 mo and life satisfaction.

Our study also has strengths. To our knowledge, this is the first study to provide insight into the association between early childhood linear growth and adult socioemotional functioning. Additional strengths include the use of data from a longitudinal cohort with ∼50 y of follow-up that include repeated measures of linear growth throughout childhood and also measures of cognitive and socioemotional functioning in adulthood with good psychometric properties. Another strength is the use of LCGA, a highly flexible statistical technique that accounts for collinearity between repeated measurements of growth and that has higher statistical power than comparable methods (25).

## Supplementary Material

nxaa337_Supplemental_File

## Notes

This work was supported by grant OPP1164115 from the Bill & Melinda Gates Foundation.

Author disclosures: The authors report no conflicts of interest.

ADS is a member of the *Journal*’s Editorial Board.

Supplemental Tables 1–15 and Supplemental Figures 1–3 are available from the “Supplementary Data” link in the online posting of the article and from the same link in the online table of contents at https://academic.oup.com/jn/.

Abbreviations used: DCCS, Dimensional Change Card Sort test; HAZ, height-for-age *z* score; INCAP, Institute of Nutrition of Central America and Panama; LCGA, latent class growth analysis; RRR, relative risk ratio.
