# Supplementary material for: Linear Growth Trajectories in Early Childhood and Adult Cognitive and Socioemotional Functioning in a Guatemalan Cohort
Source: J Nutr. 2020 Nov 26;151(1):206–13. doi: 10.1093/jn/nxaa337 (PMC7779237; doi:10.1093/jn/nxaa337)
Supplement: nxaa337_Supplemental_File [file nxaa337_supplemental_file.docx]

# **Linear growth trajectories in early childhood and adult cognitive and socioemotional functioning in a Guatemalan cohort**

María J. Ramírez-Luzuriaga, Online Supplementary Material

[**Supplemental Table 1** Fit statistics of unconditional candidate latent class growth models derived from length measures obtained at ages 0 to 84 months, by sex 2](#_Toc41485000)

[**Supplemental Table 2** Fit statistics of unconditional candidate latent class growth models derived from length measures obtained at ages 0 to 35.9 months, by sex 3](#_Toc41485001)

[**Supplemental Table 3** Fit statistics of unconditional candidate latent class growth models derived from length measures obtained at ages 36 to 84 months, by sex 4](#_Toc41485002)

[**Supplemental Table 4** Percentual distribution of children within each trajectory group over the 0-84, 0-35.9, and 36-84 months growth periods, by sex 5](#_Toc41485003)

[**Supplemental Table 5** Relative Risk Ratios (RRR) and 95% confidence intervals for predictors of growth trajectory membership at ages 0 to 35.9 months 7](#_Toc41485004)

[**Supplemental Table 6** Relative Risk Ratios (RRR) and 95% confidence intervals for predictors of growth trajectory membership at ages 36 to 84 months, by sex 8](#_Toc41485005)

[**Supplemental Table 7** Proportion of participants within each trajectory group over the 0-84-month period, without and with cognitive measures in males 9](#_Toc41485006)

[**Supplemental Table 8** Proportion of participants within each trajectory group over the 0-84-month period, without and with cognitive measures in females 9](#_Toc41485007)

[**Supplemental Table 9** Proportion of participants within each trajectory group over the 0-84-month period, without and with socioemotional measures in males 10](#_Toc41485008)

[**Supplemental Table 10** Proportion of participants within each trajectory group over the 0-84-month period, without and with cognitive measures in females 10](#_Toc41485009)

[**Supplemental Table 11** Associations of HAZ growth trajectories over the 0-35.9 and 36-84-months periods, with cognitive function at ages 40-57 years, by sex 11](#_Toc41485010)

[**Supplemental Table 12** Associations of HAZ growth trajectories over the 0-35.9 and 36-84-months periods, with socioemotional functioning at ages 40-57 years, by sex 13](#_Toc41485011)

[**Supplemental Table 13** Change in cognitive test scores (SD) per SD change in conditional growth variable, by sex 14](#_Toc41485012)

[**Supplemental Table 14** Change in socioemotional scale scores (SD) per SD change in conditional growth variable, by sex 15](#_Toc41485013)

[**Supplemental Table 15** HAZ at 24 months and adult neurodevelopmental outcomes, by sex 16](#_Toc41485014)

[**Supplemental Figure 1** Height for age-z-scores growth trajectories from birth to age 35.9 months in females (a) and males (b) 17](#_Toc41485015)

[**Supplemental Figure 2** Height for age-z-scores growth trajectories from ages 36 to 84 months in females (a) and males (b) 18](#_Toc41485016)

[**Supplemental Figure 3** Height for age-z-scores growth trajectories in children with length measures at birth, 48 months and at least one measure collected between birth and 48 months 19](#_Toc41485017)

Supplemental Table 1 Fit statistics of unconditional candidate latent class growth models derived from length measures obtained at ages 0 to 84 months, by sex

|  | Females (*n* = 722) | | |  | Males (*n* =777) | | |
| --- | --- | --- | --- | --- | --- | --- | --- |
| **Fit Statistics** | 2  Class | 3  Class | 4  Class |  | 2  Class | 3  Class | 4  Class |
|  |  |  |  |  |  |  |  |
| BIC | 16915 | 15653 | 15114 |  | 20028 | 18569 | 17976 |
|  |  |  |  |  |  |  |  |
| Entropy | 0.87 | 0.89 | 0.85 |  | 0.86 | 0.89 | 0.91 |
|  |  |  |  |  |  |  |  |
|  |  |  |  |  |  |  |  |
| LMR, *P* value | <0.001 | <0.001 | 0.10 |  | 0.04 | <0.01 | 0.35 |
|  |  |  |  |  |  |  |  |
| LRT, P value | <0.001 | <0.001 | 0.11 |  | 0.05 | <0.01 | 0.36 |
|  |  |  |  |  |  |  |  |
| BLRT, *P* value | <0.0001 | <0.0001 | <0.0001 |  | <0.0001 | <0.0001 | <0.0001 |
| Abbreviations: BIC, Bayesian Information Criterion; LMR, Lo-Mendell-Rubin Likelihood Ratio Test; LRT, Lo-Mendell-Rubin Adjusted Test; BLRT, Bootstrap Likelihood Ratio Test. | | | | | | | |

Supplemental Table 2 Fit statistics of unconditional candidate latent class growth models derived from length measures obtained at ages 0 to 35.9 months, by sex

|  | Females (*n* =556) | | |  | Males (*n* =613) | | |
| --- | --- | --- | --- | --- | --- | --- | --- |
| **Fit Statistics** | 2  Class | 3  Class | 4  Class |  | 2  Class | 3  Class | 4  Class |
|  |  |  |  |  |  |  |  |
| BIC | 11059 | 9904 | 9360 |  | 13145 | 11818 | 11220 |
|  |  |  |  |  |  |  |  |
| Entropy | 0.90 | 0.92 | 0.90 |  | 0.90 | 0.92 | 0.90 |
|  |  |  |  |  |  |  |  |
|  |  |  |  |  |  |  |  |
| LMR, *P*-value | <0.01 | <0.01 | 0.15 |  | <0.001 | 0.04 | <0.01 |
|  |  |  |  |  |  |  |  |
|  |  |  |  |  |  |  |  |
| LRT, P-value | <0.01 | <0.01 | 0.16 |  | <0.001 | 0.05 | <0.01 |
|  |  |  |  |  |  |  |  |
|  |  |  |  |  |  |  |  |
| BLRT, *P* value | <0.0001 | <0.0001 | <0.0001 |  | <0.0001 | <0.0001 | <0.0001 |
| Abbreviations: BIC, Bayesian Information Criterion; LMR, Lo-Mendell-Rubin Likelihood Ratio Test; LRT, Lo-Mendell-Rubin Adjusted Test; BLRT, Bootstrap Likelihood Ratio Test. | | | | | | | |

Supplemental Table 3 Fit statistics of unconditional candidate latent class growth models derived from length measures obtained at ages 36 to 84 months, by sex

|  | Females (*n* =425) | | |  | Males (*n* =476) | | |
| --- | --- | --- | --- | --- | --- | --- | --- |
| **Fit Statistics** | 2  Class | 3  Class | 4  Class |  | 2  Class | 3  Class | 4  Class |
|  |  |  |  |  |  |  |  |
| BIC | 4368 | 3605 | 3153 |  | 4654 | 3889 | 3472 |
|  |  |  |  |  |  |  |  |
| Entropy | 0.85 | 0.92 | 0.92 |  | 0.86 | 0.92 | 0.92 |
|  |  |  |  |  |  |  |  |
|  |  |  |  |  |  |  |  |
| LMR, *P*-value | 0.04 | 0.08 | 0.12 |  | 0.02 | <0.01 | 0.12 |
|  |  |  |  |  |  |  |  |
|  |  |  |  |  |  |  |  |
| LRT, P-value | 0.05 | 0.08 | 0.13 |  | 0.02 | <0.01 | 0.13 |
|  |  |  |  |  |  |  |  |
|  |  |  |  |  |  |  |  |
| BLRT, *P* value | <0.0001 | <0.0001 | <0.0001 |  | <0.0001 | <0.0001 | <0.0001 |
| Abbreviations: BIC, Bayesian Information Criterion; LMR, Lo-Mendell-Rubin Likelihood Ratio Test; LRT, Lo-Mendell-Rubin Adjusted Test; BLRT, Bootstrap Likelihood Ratio Test. | | | | | | | |

Supplemental Table 4 Percentual distribution of children within each trajectory group over the 0-84, 0-35.9, and 36-84 months growth periods, by sex

1. Females

|  | | **Trajectories derived from length measures obtained at ages 0-35.9 months** | | | **Total** |
| --- | --- | --- | --- | --- | --- |
|  |  | High, n (%) | Intermediate, n (%) | Low n, (%) |  |
| **Trajectories derived from length measures obtained at ages 0-84 months** | High | 168 (30.2) | 37 (6.7) | 0 (0.0) | 205 (36.9) |
|  | Intermediate | 2 (0.4) | 223 (40.1) | 35 (6.3) | 260 (46.8) |
|  | Low | 0 (0.0) | 3 (0.5) | 88 (15.8) | 91 (16.4) |
| **Total** | | 170 (30.6) | 263 (47.3) | 1. (22.1) | 556 (100) |

|  | | **Trajectories derived from length measures obtained at ages 36-84 months** | | **Total** |
| --- | --- | --- | --- | --- |
|  |  | High, n (%) | Low, n (%) |  |
| **Trajectories derived from length measures obtained at ages 0-84 months** | High | 123 (28.9) | 0 (0.0) | 123 (28.9) |
|  | Intermediate | 125 (29.4) | 103 (24.2) | 228 (53.6) |
|  | Low | 0 (0.0) | 74 (17.4) | 74 (17.4) |
| **Total** | | 248 (58.3) | 177 (41.6) | 425 (100) |

1. Males

|  | | **Trajectories derived from length measures obtained at ages 0-35.9 months** | | | **Total** |
| --- | --- | --- | --- | --- | --- |
|  |  | High, n (%) | Intermediate, n (%) | Low, n (%) |  |
| **Trajectories derived from length measures obtained at ages**  **0-84 months** | High | 180 (29.4) | 26 (4.2) | 0 (0.0) | 206 (33.6) |
|  | Intermediate | 4 (0.65) | 264 (43.1) | 24 (3.9) | 292 (47.6) |
|  | Low | 0 (0.0) | 9 (1.5) | 106 (17.3) | 115 (18.8) |
| **Total** | | 184 (30.0) | 299 (48.8) | 130 (21.2) | 613 (100) |

|  | | **Trajectories derived from length measures obtained at ages 36-84 months** | | | **Total** |
| --- | --- | --- | --- | --- | --- |
|  |  | High, n (%) | Intermediate, n (%) | Low, n (%) |  |
| **Trajectories derived from length measures obtained at ages 0-84 months** | High | 104 (21.8) | 25 (5.2) | 0 (0.0) | 129 (27.1) |
|  | Intermediate | 14 (2.9) | 204 (42.9) | 33 (6.9) | 251 (52.7) |
|  | Low | 0 (0.0) | 12 (2.5) | 84 (17.6) | 96 (20.2) |
| **Total** | | 118 (24.9) | 241 (50.6) | 117 (24.5) | 476 (100) |

Supplemental Table 5 Relative Risk Ratios (RRR) and 95% confidence intervals for predictors of growth trajectory membership at ages 0 to 35.9 months

|  | **Intermediate- vs**  **low- growth trajectory** | **High- vs.**  **low-growth trajectory** |
| --- | --- | --- |
|  | **RRR (95% CI)** | **RRR (95% CI)** |
| Childhood socioeconomic tertile |  |  |
| Middle vs. poorest | 1.02 (0.66, 1.57) | 1.14 (0.66, 1.97) |
| Wealthiest vs. poorest | 1.48 (0.91, 2.40) | **2.03 (1.11, 3.69)** |
| Mother’s age at childbirth | 0.99 (0.96, 1.02) | 1.01 (0.98, 1.04) |
| Mother’s years of schooling | 0.93 (0.81, 1.06) | 0.96 (0.82, 1.13) |
| Mother’s height | **1.10 (1.06, 1.15)** | **1.20 (1.14, 1.26)** |
| Sample size was 1169. Estimates adjusted for fixed effects of birth village, birth year and sex. For missing covariates, we used multiple imputation techniques. Confidence intervals account for clustering at the mother level. | | |

Supplemental Table 6 Relative Risk Ratios (RRR) and 95% confidence intervals for predictors of growth trajectory membership at ages 36 to 84 months, by sex

|  | **Intermediate- vs**  **low-growth trajectory ^a^** | | | **High- vs.**  **low-growth trajectory** | |
| --- | --- | --- | --- | --- | --- |
|  | Females | Males  (n=476) | Females  (n=425) | | Males  (n=476) |
| Exposure to *Atole* in full first 1,000 days. | - | 2.21 (0.84, 5.81) | **2.99 (1.26, 7.11)** | | 1.65 (0.52, 5.20)^b^ |
| Childhood socioeconomic tertile |  |  |  | |  |
| Middle vs. poorest | - | 1.13 (0.60, 2.13) | 1.37 (0.76, 2.43) | | 1.03 (0.45, 2.32) |
| Wealthiest vs. poorest | - | 1.82 (0.92, 3.58) | 1.51 (0.81, 2.82) | | 2.27 (0.93, 5.51) |
| Mother’s age at childbirth | - | 0.99 (0.96, 1.03) | 0.98 (0.95, 1.02) | | 1.04 (0.99, 1.09) |
| Mother’s years of schooling | - | 0.96 (0.90, 1.16) | 0.98 (0.84, 1.14) | | 0.98 (0.78, 1.22) |
| Mother’s height | - | **1.12 (1.05, 1.20)** | **1.17 (1.10, 1.23)** | | **1.23 (1.13, 1.34)** |
| Estimates adjusted for fixed effects of birth village and birth year. For missing covariates, we used multiple imputation techniques. Confidence intervals account for clustering at the mother level.  ^a^ Because only two growth trajectories (high and low) were identified at ages 36 to 84 months in females, no results are reported for intermediate-vs low growth trajectory.  ^b^ p=0.43 for equality of parameter estimates across sexes. | | | | | |

Supplemental Table 7 Proportion of participants within each trajectory group over the 0-84-month period, without and with cognitive measures in males

|  | **Raven,**  **n (%)** | | **Working**  **Memory, n (%)** | | **Inhibitory**  **Control, n (%)** | | **Cognitive**  **Flexibility, n (%)** | | **N Total** |
| --- | --- | --- | --- | --- | --- | --- | --- | --- | --- |
|  | **Without** | **With** | **Without** | **With** | **Without** | **With** | **Without** | **With** |  |
| **High trajectory** | 117 (32.9) | 132 (31.2) | 134 (34.6) | 115 (29.5) | 133 (34.5) | 116 (29.7) | 132 (34.5) | 117 (29.6) | 249 |
| **Intermediate trajectory** | 178 (50.1) | 210 (49.8) | 190 (49.1) | 198 (50.8) | 190 (49.2) | 198 (50.6) | 190 (49.7) | 198 (50.1) | 388 |
| **Low trajectory** | 60 (16.9) | 80 (18.9) | 63 (16.3) | 77 (19.7) | 63 (16.3) | 77 (19.7) | 60 (15.7) | 80 (20.2) | 140 |
| **Total** | 355 (100) | 422 (100) | 387 (100) | 390 (100) | 386 (100) | 391 (100) | 382 (100) | 395 (100) | 777 |
| **χ ^2^ *p-value*** | 0.73 | | 0.22 | | 0.26 | | 0.16 | |  |

Supplemental Table 8 Proportion of participants within each trajectory group over the 0-84-month period, without and with cognitive measures in females

|  | **Raven,**  **n (%)** | | **Working**  **Memory, n (%)** | | **Inhibitory**  **Control, n (%)** | | **Cognitive**  **Flexibility, n (%)** | | **N Total** |
| --- | --- | --- | --- | --- | --- | --- | --- | --- | --- |
|  | **Without** | **With** | **Without** | **With** | **Without** | **With** | **Without** | **With** |  |
| **High trajectory** | 75 (37.7) | 173 (33.1) | 95 (37.4) | 153 (32.7) | 94 (37.1) | 154 (32.8) | 92 (37.2) | 156 (32.8) | 248 |
| **Intermediate trajectory** | 89 (44.7) | 272 (52.0) | 119 (46.8) | 242 (51.7) | 117 (46.2) | 244 (52.0) | 114 (46.1) | 247 (52.0) | 361 |
| **Low trajectory** | 35 (17.6) | 78 (14.9) | 40 (15.7) | 73 (15.6) | 42 (16.6) | 71 (15.1) | 41 (16.6) | 72 (15.2) | 113 |
| **Total** | 199 (100) | 523 (100) | 254 (100) | 468 (100) | 253 (100) | 469 (100) | 247 (100) | 475 (100) | 722 |
| **χ ^2^ *p-value*** | 0.21 | | 0.40 | | 0.33 | | 0.33 | |  |

Supplemental Table 9 Proportion of participants within each trajectory group over the 0-84-month period, without and with socioemotional measures in males

|  | **Happiness,**  **n (%)** | | **Life satisfaction,**  **n (%)** | | **Meaning and purpose, n (%)** | | **Self-efficacy,**  **n (%)** | | **N Total** |
| --- | --- | --- | --- | --- | --- | --- | --- | --- | --- |
|  | **Without** | **With** | **Without** | **With** | **Without** | **With** | **Without** | **With** |  |
| **High trajectory** | 131 (34.5) | 118 (29.7) | 131(34.5) | 118 (29.7) | 131 (34.4) | 118 (29.8) | 131 (34.4) | 118 (29.8) | 249 |
| **Intermediate trajectory** | 187 (49.2) | 201 (50.6) | 187 (49.2) | 201 (50.6) | 188 (49.3) | 200 (50.5) | 188 (49.3) | 200 (50.5) | 388 |
| **Low trajectory** | 62 (16.3) | 78 (19.6) | 62 (16.3) | 78 (19.6) | 62 (16.3) | 78 (19.7) | 62 (16.3) | 78 (19.7) | 140 |
| **Total** | 380 (100) | 397 (100) | 380 (100) | 397 (100) | 381 (100) | 396 (100) | 381 (100) | 396 (100) | 777 |
| **χ ^2^ *p-value*** | 0.27 | | 0.26 | | 0.27 | | 0.27 | |  |

Supplemental Table 10 Proportion of participants within each trajectory group over the 0-84-month period, without and with cognitive measures in females

|  | **Happiness,**  **n (%)** | | **Life satisfaction,**  **n (%)** | | **Meaning and purpose, n (%)** | | **Self-efficacy,**  **n (%)** | | **N Total** |
| --- | --- | --- | --- | --- | --- | --- | --- | --- | --- |
|  | **Without** | **With** | **Without** | **With** | **Without** | **With** | **Without** | **With** |  |
| **High trajectory** | 88 (37.6) | 160 (32.8) | 88 (37.4) | 160 (32.8) | 89 (37.7) | 159 (32.7) | 88 (37.3) | 160 (32.9) | 248 |
| **Intermediate trajectory** | 112 (47.9) | 249 (51.0) | 113 (48.1) | 248 (50.9) | 113 (47.9) | 248 (51.0) | 113 (47.9) | 248 (51.0) | 361 |
| **Low trajectory** | 34 (14.5) | 79 (16.2) | 34 (14.5) | 79 (16.2) | 34 (14.4) | 79 (16.3) | 35 (14.8) | 78 (16.0) | 113 |
| **Total** | 234 (100) | 488 (100) | 235 (100) | 487 (100) | 236 (100) | 486 (100) | 236 (100) | 486 (100) | 722 |
| **χ ^2^ *p-value*** | 0.43 | | 0.46 | | 0.40 | | 0.51 | |  |

Supplemental Table 11 Associations of HAZ growth trajectories over the 0-35.9 and 36-84-months periods, with cognitive function at ages 40-57 years, by sex

|  | **0 to 35.9 months ^a^** | |  | **36 to 84 months ^b^** | |
| --- | --- | --- | --- | --- | --- |
|  | **Intermediate- vs low-growth trajectory** | **High- vs low-growth trajectory** |  | **Intermediate- vs low-growth trajectory ^c^** | **High- vs low-growth trajectory** |
| **Non-verbal fluid intelligence** |  |  |  |  |  |
| Women | 0.66 (-0.63, 1.95) | 0.94 (-0.57, 2.45) |  | - | -0.72 (-2.03,0.59) |
| Men | **3.37 (0.88, 3.86)** | **4.53 (2.75, 6.31)** |  | **1.95 (0.47, 3.44)** | **2.76 (0.48, 5.03)** |
| **Working memory** |  |  |  |  |  |
| Women | 0.52 (-0.52, 1.55) | 0.96 (-0.37, 2.30) |  | - | 0.58 (-0.36, 1.53) |
| Men | 0.84 (-0.27, 1.96) | **1.71 (0.31, 3.11)** |  | 0.95 (-0.12, 2.02) | 0.76 (-0.79, 2.31) |
| **Inhibitory control** |  |  |  |  |  |
| Women | **0.35 (0.40, 0.65)** | 0.29 (-0.06, 0.65) |  | - | -0.05 (-0.33, 0.23) |
| Men | **0.50 (0.19, 0.81)** | **0.68 (0.28, 1.07)** |  | 0.09 (-0.28, 0.46) | 0.28 (0.15, 0.71) |
| **Cognitive flexibility** |  |  |  |  |  |
| Women | 0.09 (-0.44, 0.62) | 0.19 (-0.43, 0.82) |  | - | 0.26 (-0.23, 0.76) |
| Men | **0.73 (0.19, 1.26)** | **1.14 (0.58, 1.70)** |  | **0.65 (0.04, 1.27)** | 0.54 (-0.22, 1.29) |

^a^ For women and men respectively, sample sizes were 404 and 336 for Raven’s Progressive Matrices (non-verbal fluid intelligence), 368 and 310 for List Sorting Working Memory, 370 and 312 for Flanker Inhibitory Control and Attention, 372 and 314 for Dimensional Change Card Sort (Cognitive flexibility).

^b^ For women and men respectively, sample sizes were 318 and 265 for Raven’s Progressive Matrices (non-verbal fluid intelligence), 285 and 250 for List Sorting Working Memory, 283 and 250 for Flanker Inhibitory Control and Attention, 287 and 253 for Dimensional Change Card Sort (Cognitive flexibility).

^c^ Because only two growth trajectories (high and low) were identified at ages 36 to 84 months in females, no results are reported for intermediate-vs low growth trajectory.

All models adjusted for fixed effects of birth village, birth year, exposure to supplementation from conception to age 2 years, the interaction term specifying exposure to *atole* from conception to age two years, maternal age at childbirth and maternal height (log-transformed), maternal schooling and household socioeconomic status in 1967-75. For missing covariates, we used multiple imputation techniques. Confidence intervals account for clustering at the household level.

Supplemental Table 12 Associations of HAZ growth trajectories over the 0-35.9 and 36-84-months periods, with socioemotional functioning at ages 40-57 years, by sex

|  | **0 to 35.9 months ^a^** | |  | **36 to 84 months ^b^** | |
| --- | --- | --- | --- | --- | --- |
|  | **Intermediate- vs low-growth trajectory** | **High- vs low-growth trajectory** |  | **Intermediate- vs low-growth trajectory ^c^** | **High- vs low-growth trajectory** |
| **Happiness** |  |  |  |  |  |
| Women | 0.10 (-0.18, 0.39) | **0.34 (0.02, 0.67)** |  | - | -0.08 (-0.35, 0.19) |
| Men | 0.19 (-0.08, 0.46) | 0.03 (-0.27, 0.33) |  | 0.24 (-0.02, 0.51) | 0.27 (-0.04, 0.59) |
| **Life Satisfaction** |  |  |  |  |  |
| Women | 0.85 (-0.14, 1.86) | 0.85 (-0.33, 2.04) |  | - | 0.31 (-0.54, 1.16) |
| Men | 0.77 (-0.35, 1.90) | 0.04 (-1.24, 1.33) |  | -0.15 (-1.29, 0.97) | 0.47 (-1.01, 1.95) |
| **Meaning and Purpose** |  |  |  |  |  |
| Women | **1.25 (0.13, 2.36)** | 0.99 (-0.25, 2.24) |  | - | 0.38 (-0.71, 1.47) |
| Men | **1.39 (0.21, 2.57)** | **1.82 (0.47, 3.17)** |  | 0.03 (-1.28, 1.35) | 0.04 (-1.54, 1.63) |
| **Self-efficacy** |  |  |  |  |  |
| Women | 1.58 (-0.36, 3.53) | 1.25 (-1.03, 3.56) |  |  | 0.74 (1.12, 2.61) |
| Men | 0.24 (-1.71, 2.20) | -1.31 (-3.57, 0.95) |  | 0.04 (-2.10, 2.20) | 0.98 (-1.61, 3.58) |

^a^ For women and men respectively, sample sizes were 382 and 312 for Subjective Happiness scale, 381 and 312 for NIH Toolbox Life Satisfaction scale, 380 and 312 for NIH Toolbox Meaning and purpose scale and NIH Toolbox Self-efficacy scale.

^b^ For women and men respectively, sample sizes were 297 and 258 for Subjective Happiness scale, 297 and 258 for NIH Toolbox Life Satisfaction scale, 297 and 257 for NIH Toolbox Meaning and purpose scale and NIH Toolbox Self-efficacy scale.

^c^ Because only two growth trajectories (high and low) were identified at ages 36 to 84 months in females, no results are reported for intermediate-vs low growth trajectory.

All models adjusted for fixed effects of birth village, birth year, exposure to supplementation from conception to age 2 years, the interaction term specifying exposure to *atole* from conception to age two years, maternal age at childbirth and maternal height (log-transformed), maternal schooling and household socioeconomic status in 1967-75. For missing covariates, we used multiple imputation techniques. Confidence intervals account for clustering at the household level.

Supplemental Table 13 Change in cognitive test scores (SD) per SD change in conditional growth variable, by sex

|  | **Non-verbal fluid intelligence** | **Working memory** | **Inhibitory control** | **Cognitive flexibility** |
| --- | --- | --- | --- | --- |
| **Birth length Z score** |  |  |  |  |
| Women | 0.01 (-0.15, 0.17) | 0.04 (-0.12, 0.21) | 0.12 (-0.04, 0.28) | -0.01 (-0.20, 0.17) |
| Men | **0.16 (0.04, 0.28)** | 0.07 (-0.08, 0.22 | **0.17 (0.01, 0.32)** | **0.20 (0.08, 0.31)** |
| Pooled | 0.08 (-0.02, 0.18) | 0.06 (-0.05, 0.17) | **0.13 (0.03, 0.24**) | 0.10 (-0.01, 0.20) |
| **Conditional length at 24 months** |  |  |  |  |
| Women | -0.12 (-0.32, 0.08) | -0.02 (-0.22, 0.17) | -0.04 (-0.23, 0.15) | 0.02 (-0.21, 0.25) |
| Men | **0.24 (0.03, 0.45)** | 0.18 (-0.06, 0.42) | 0.01 (-0.22, 0.23) | 0.15 (-0.02, 0.33) |
| Pooled | 0.04 (-0.09, 0.18) | 0.08(-0.06, 0.22) | 0.01 (-0.11, 0.14) | 0.12 (-0.02, 0.25) |
| **Conditional length at 48 months** |  |  |  |  |
| Women | -0.09 (-0.31, 0.12) | -0.11 (-0.31, 0.10) | -0.07 (-0.25, 0.12) | -0.06 (-0.28, 0.15) |
| Men | -0.05 (-0.19, 0.09) | -0.05 (-0.35, 0.24) | 0.01 (-0.22, 0.25) | -0.09 (-0.20, 0.02) |
| Pooled | -0.08 (-0.20, 0.05) | -0.06 (-0.22, 0.10) | -0.01(-0.15, 0.13) | -0.10 (-0.21, 0.01) |

Data are beta coefficients (95% Confidence Intervals (CI)) from linear regression models. Sample sizes in women and men respectively are 128 and 105 for non-verbal fluid intelligence, 119 and 100 for working memory, 120 and 100 for inhibitory control, 118 and 102 for cognitive flexibility. Estimates adjusted for maternal age at childbirth and maternal height (log-transformed), maternal education, socioeconomic status in 1967-75, fixed effects of birth village, birth year, exposure to supplementation from conception to age two y, and the interaction term specifying exposure to *atole* from conception to age two y. Pooled models adjusted for sex. For missing covariates, we used multiple imputation techniques. Confidence intervals account for clustering at the household level.

Supplemental Table 14 Change in socioemotional scale scores (SD) per SD change in conditional growth variable, by sex

|  | **Happiness** | **Life satisfaction** | **Meaning and purpose** | **Self-efficacy** |
| --- | --- | --- | --- | --- |
| **Birth length Z score** |  |  |  |  |
| Women | 0.06 (-0.13, 0.24) | 0.14 (-0.03, 0.32) | 0.12 (-0.02, 0.25) | 0.04 (-0.15, 0.23) |
| Men | 0.14 (-0.02, 0.31) | **0.18 (0.01, 0.34)** | 0.12 (-0.02, 0.26) | -0.06 (-0.18, 0.06) |
| Pooled | 0.11(-0.01, 0.24) | **0.17 (0.05, 0.28**) | **0.10 (0.00, 0.19)** | -0.01 (-0.12, 0.09) |
| **Conditional length at 24 months** |  |  |  |  |
| Women | 0.15 (-0.03, 0.34) | 0.01 (-0.17, 0.19) | -0.19 (-0.39, 0.01) | -0.03 (-0.23, 0.17) |
| Men | -0.01 (-0.19, 0.17) | -0.15 (-0.35, 0.07) | 0.05 (-0.18, 0.29) | -0.02 (-0.23, 0.19) |
| Pooled | 0.06 (-0.06, 0.18) | -0.05 (-0.18, 0.08) | -0.03 (-0.18, 0.12) | -0.04 (-0.19, 0.10) |
| **Conditional length at 48 months** |  |  |  |  |
| Women | -0.09 (-0.32, 0.13) | -0.16 (-0.39, 0.06) | -0.14 (-0.35, 0.06) | -0.04 (-0.27, 0.17) |
| Men | **0.12 (0.01, 0.23)** | -0.13 (-0.27, 0.01) | -0.11 (-0.26, 0.05) | -0.11 (-0.27, 0.04) |
| Pooled | 0.03 (-0.08, 0.16) | **-0.14 (-0.26, -0.02)** | -0.11 (-0.22, -0.01) | -0.08 (-0.21, 0.05) |

Data are beta coefficients (95% Confidence Intervals (CI)) from linear regression models. Sample size for all outcomes is 121 in women and 100 in men. Estimates adjusted for maternal age at childbirth and maternal height (log-transformed), maternal education, socioeconomic status in 1967-75, fixed effects of birth village, birth year, exposure to supplementation from conception to age two y, and the interaction term specifying exposure to *atole* from conception to age two y. Pooled models adjusted for sex. For missing covariates, we used multiple imputation techniques. Confidence intervals account for clustering at the household level.

Supplemental Table 15 HAZ at 24 months and adult neurodevelopmental outcomes, by sex

|  | **Pooled** | **Women** | **Men** |
| --- | --- | --- | --- |
| **Cognitive ability** |  |  |  |
| Non-verbal fluid intelligence | **0.61 (0.26, 0.96)** | 0.34 (-0.07, 0.75) | **0.93 (0.39, 1.48)** |
| Working memory | **0.55 (0.29, 0.80)** | **0.59 (0.25, 0.93)** | **0.51 (0.15, 0.87)** |
| Inhibitory control | **0.14 (0.06, 0.21)** | 0.04 (-0.05, 0.13) | **0.24 (0.13, 0.34)** |
| Cognitive flexibility | **0.24 (0.11, 0.36)** | 0.13 (-0.05, 0.30) | **0.35 (0.18, 0.51)** |
| **Socioemotional functioning** |  |  |  |
| Happiness | 0.05 (-0.00, 0.11) | **0.08 (0.00, 0.17)** | 0.02 (-0.07, 0.10) |
| Life Satisfaction | 0.13 (-0.09, 0.36) | 0.18 (-0.11, 0.48) | 0.07 (-0.25, 0.38) |
| Meaning and purpose | **0.27 (0.02, 0.52)** | 0.19 (-0.16, 0.53) | 0.33 (-0.04, 0.70) |
| Self-efficacy | 0.15 (-0.26, 0.57) | 0.47 (-0.12, 1.06) | -0.16 (-0.77, 0.45) |

Data are beta coefficients (95% CI) from linear regression models. Sample size is 647 and 515 for non-verbal fluid intelligence, 579 and 478 for working memory, 580 and 477 for inhibitory control, and 589 and 482 for cognitive flexibility, 607 and 487 for happiness, 606 and 487 for life satisfaction, 605 and 486 for meaning and purpose, and 605 and 486 for self-efficacy in women and men, respectively.

Estimates adjusted for maternal age at childbirth and maternal height (log-transformed), maternal education, socioeconomic status in 1967-75, fixed effects of birth village, birth year, exposure to supplementation from conception to age two y, and the interaction term specifying exposure to *atole* from conception to age two y. Pooled models adjusted for sex. For missing covariates, we used multiple imputation techniques. Confidence intervals account for clustering at the household level.

Supplemental Figure 1 Height for age-z-scores growth trajectories from birth to age 35.9 months in females (a) and males (b)

**b)**

High trajectory (30.0%, n=184)

Intermediate trajectory (48.8%, n=299)

Low trajectory (21.2%, n=130)

Supplemental Figure 2 Height for age-z-scores growth trajectories from ages 36 to 84 months in females (a) and males (b)

**a)**

**b)**

High trajectory (24.8%, n=118)

Intermediate trajectory (50.6%, n=241)

Low trajectory (24.6%, n=117)

Supplemental Figure 3 Height for age-z-scores growth trajectories in children with length measures at birth, 48 months and at least one measure collected between birth and 48 months

High trajectory (32.3 %, n=92)

Intermediate trajectory (49.8%, n=142)

Low trajectory (17.9%, n=51)
